# THE IMPACT OF SYNCHRONIZED DYADIC DANCE ON SOCIAL BONDING AND INTERGROUP CLOSENESS

**DOI:** 10.1093/geroni/igae098.0606

**Published:** 2024-12-31

**Authors:** Assaf Suberry, Ehud Bodner

**Affiliations:** School of Social Work, Bar-Ilan University, Saad, HaDarom, Israel; Bar-Ilan University, Ramat Gan, HaMerkaz, Israel

## Abstract

Synchronization of bodies in time encourages positive social functions such as cooperation and compassion. This study examined the effect of synchronous dance movements on social bonding and perceived intergenerational closeness. 168 participants ranging in age from 20 to 45 (young adults) and 65+ (older adults) were randomly assigned to six dyad conditions, which included dancing synchronously or asynchronously with an in-age-group or with an out-age-group stranger for 11 minutes. Participants completed social bonding with the partner and perception of distance between social groups (in terms of religiosity, ethnographic origin, political stance, and age) questionnaires. We used generalized estimating equations (GEE) modeling to assess variation across both individual measurements and measurements within dyads. Social bonding significantly increased in the synchronized condition (b = 0.51, p =.014), with participants reporting higher bonding with the dancing partner compared to the asynchronous condition. Relative to older participants, younger participants demonstrated a greater enhancement in perceived closeness across diverse social groups, as well as a greater increase in perceived closeness between age groups following the intervention. Lastly, participants reported a greater sense of closeness across generations when dancing asynchronously with in-group members compared to out-group members. We explore the possibility that declining openness and a shift towards conservatism in older adults may influence social connectedness and attitudes. Intergenerational dance programs may promote intergenerational relations and social cohesion, particularly for young adults. Practitioners from various fields - welfare, nursing, sport, music education, and community therapy may benefit from intergenerational rhythmic activity.

